# Autophagy and Macropinocytosis: Keeping an Eye on the Corneal/Limbal Epithelia

**DOI:** 10.1167/iovs.16-21111

**Published:** 2017-01

**Authors:** Han Peng, Jong Kook Park, Robert M. Lavker

**Affiliations:** Department of Dermatology, Feinberg School of Medicine, Northwestern University, Chicago, Illinois, United States

**Keywords:** autophagy, macropinocytosis, limbal epithelium, corneal epithelium

## Abstract

Autophagy and macropinocytosis are processes that are vital for cellular homeostasis, and help cells respond to stress and take up large amounts of material, respectively. The limbal and corneal epithelia have the machinery necessary to carry out both processes; however, autophagy and macropinocytosis are relatively understudied in these two epithelia. In this Perspectives, we describe the basic principles behind macropinocytosis and autophagy, discuss how these two processes are regulated in the limbal and corneal epithelia, consider how these two processes impact on the physiology of limbal and corneal epithelia, and elaborate on areas of future research in autophagy and macropinocytosis as related to the limbal/corneal epithelia.

The term “autophagy” is attributed to the venerable morphologist and cell biologist Christian de Duve.^1^ However, this term has been in the scientific literature as early as 1860 (see the literature of Karaqnasios and Ktistakis^2^). Nonetheless, Christian de Duve coined the term “autophagic vacuoles” to describe the self-eating function of lysosomes,^1^ which still is the operative definition used today. Christian de Duve won the Nobel Prize in Physiology and Medicine in 1974 for the discovery of the lysosome, and the same prize was awarded in 2016 to Yoshinori Ohsumi for his work in yeast identifying genes essential for autophagy.^3^ Ohsumi's work has led to our current understanding that autophagy regulates numerous physiologic processes where cellular components must be degraded and recycled. Examples of such processes are the cell's response to starvation and other stresses,^4^ removal of bacteria and viruses,^5^ elimination of damaged proteins and organelles that occur during aging,^6^ and maintenance of stem cell homeostasis.^7^ Given the numerous ways that autophagy impacts cellular homeostasis, it is not remarkable that this cellular process has been implicated in ocular health and disease (see prior reviews^8,9^). Surprisingly, scant attention has been directed at dissecting autophagy in the corneal and limbal epithelia.

Macropinocytosis was described first by Lewis in 1931^10^ and enables cells to nonselectively engulf and take up large volumes of fluid and membrane via the closure of plasma membrane protrusions.^11^ In addition to enabling cells to “gulp” large amounts of fluids, macropinocytosis is now recognized as a means of pathogen egress into cells as well as having important roles in tumorigenesis and cancer therapeutics.^12^ Similar to autophagy, macropinocytosis has been markedly understudied in the corneal/limbal epithelia. We offer a view of how the regulation of autophagy and macropinocytosis differs between limbal and corneal epithelia, and discuss areas that are ripe for future investigation.

## Autophagy

**Figure 1:**
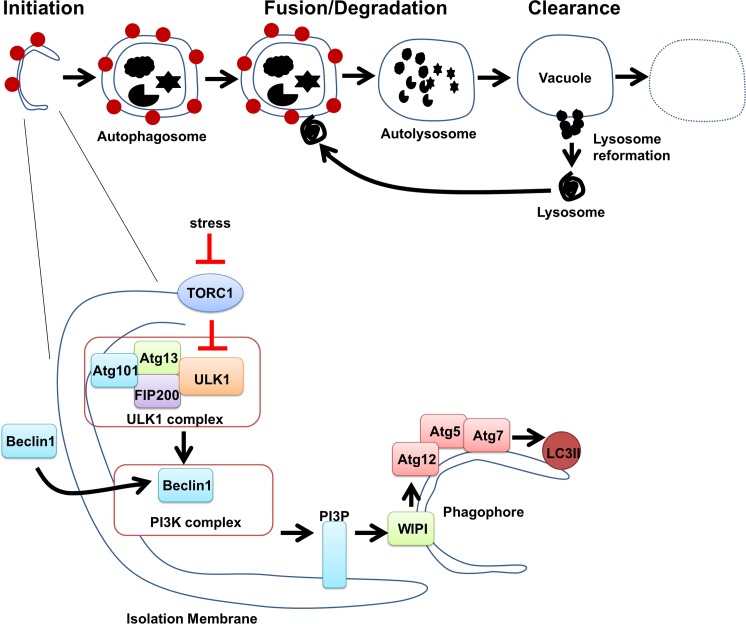
A schematic representation of the stages of autophagy. Autophagy begins with the formation of the phagophore (initiation stage). The expansion of the phagophore results in an autophagosome. Autophagosomes can engulf cytoplasmic materials. When an autophagosome fuses with a lysosome, it forms an autolysosome where the sequestered material is degraded in autolyosome (fusion/degradation stage). Finally, autolysosome is recycled to form new lysosome (late stages).

Autophagy is a highly evolutionary conserved cellular process by which cytoplasmic material (e.g., mitochondria, Golgi, nuclei) is segregated into double membrane vesicles (autophagosomes) that fuse with lysosomes for degradation.^13,14^ Autophagy can be divided into early and late stages (Fig. 1). The early stages involve the formation of a phagophore (autophagosome) via the sequential activation of a series of complexes, which results in the formation of an isolation membrane that ultimately engulfs the cytoplasmic material to be digested. Initiation starts with a stress signal, releasing the inhibitory effects of the mechanistic target of rapamycin (mTOR) on the UNC51-like kinase (ULK1), which then phosphorylates its substrates Atg13, Fip200, and Atg101 (Fig. 1). Nucleation involves the recruitment of Beclin-1, which ultimately results in recruitment of WIPI via PtdIns3P leading to the formation of a nascent phagophore. The last stage is autophagosome elongation and closure involving Atg12 conjugation to Atg5 via Atg7. This complex facilitates the lipidation of LC3I to LC3II onto forming double-membrane autophagosomes. Ultimately, the autophagosome fuses with a lysosome forming the autolysosome, which degrades the ingested material. Until recently, most of the research on autophagy has focused on delineating these early stages.^15^ Less attention has been directed toward understanding the late stages of autophagy, which involve autolysosome clearance and lysosome reformation.^15,16^

## Macropinocytosis

**Figure 2:**
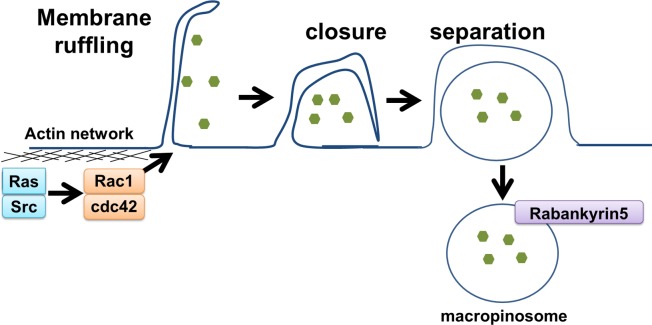
A schematic representation of macropinocytosis. Ras and Src activates Rac1 and cdc42, leading to actin cytoskeleton rearrangement at the plasma membrane and consequently membrane ruffling. Ruffles may close and trap bulk fluid. Maturation of macropinosomes involve recruitment of rabankyrin5.

Macropinocytosis is an endocytic process resulting in the formation of large (0.2–2 μm) macropinosomes (see the reports of Lim and Gleeson,^11^ and Maltese and Overmeyer,^17^ and references therein). Macropinocytosis is initiated as a response to growth factor stimulation, such as epidermal growth factor (EGF), platelet-derived growth factor (PDGF), or the tumor promotor tissue plasminogen activator (TPA).^11,18,19^ Such stimulation results in actin-mediated membrane ruffling (lamellipodia) at the plasma membrane (Fig. 2). Most lamellipodia retract back into the cell; however, a subset fold back upon themselves and fuse with the membrane, which generates large vesicles termed macropinosomes.^11^ Membrane ruffling with its associated remodeling of the cytoskeleton appears to be required for macropinocytosis, but not sufficient for macropinosome formation (Fig. 2).^20,21^ Once formed, macropinosomes undergo a maturation process, are either degraded via a late endosome/lysosome process, or recycled back to the plasma membrane.^11^ Precise signaling events and how components of macropinocytosis are coordinated are unclear; however, macropinocytosis is likely to be distinctive in different cell types.^11,12,17^ When the process of macropinocytosis becomes dysregulated, one of the morphologic features is the appearance of large cytoplasmic vacuoles.^17,22^ Recently, there has been renewed interest in macropincytosis due to the observations that cancer cells possess increased macropinocytotic activity to enhance metabolism, signal transduction, and metastasis (see the report of Ha et al.^12^ and references therein). Consequently, there has been high interest in the use of macropinocytosis for targeted therapy development using macropinocytosing antibodies.^23^ Given that varying cell types can have wide ranges in macropinocytotic activity,^11,12,17^ determining how macropinocytosis is regulated in limbal and corneal epithelia seems warranted.

## Corneal/Limbal Epithelia: Distinctions Provide Insights

**Figure 3:**
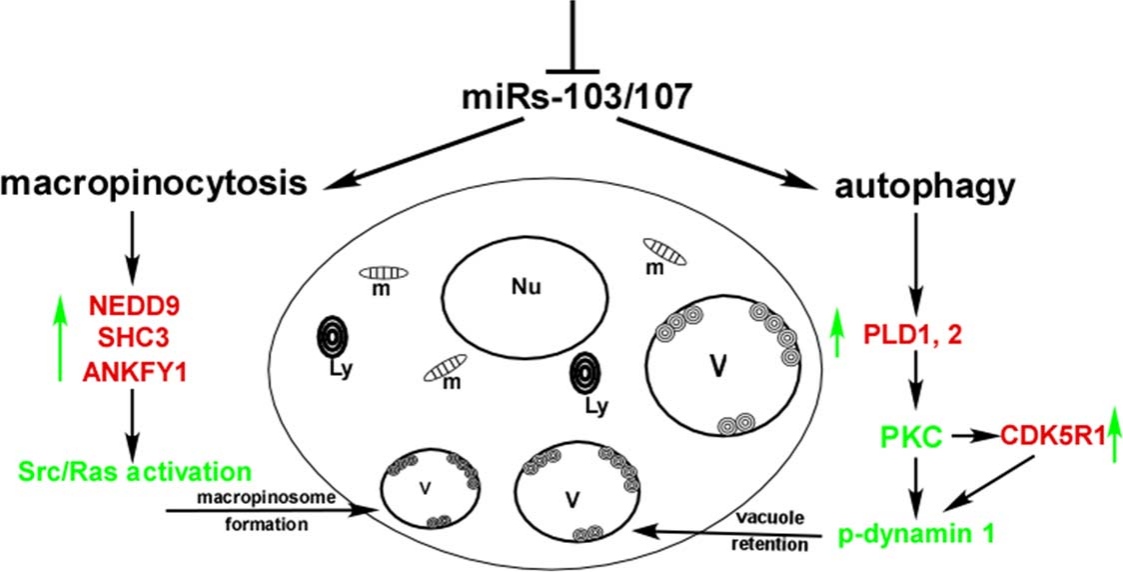
A schematic representation of how miRs-103/107 coordinately regulate aspects of both macropinocytosis and autophagy. Loss of miRs-103/107 has two effects: (1) Such loss upregulates macropinocytosis via targeting *NEDD9*, *SHC3*, and *ANKFY1*, which collectively activates Src/Ras. This yields numerous vacuoles. (2) Such loss upregulates PLD1 and PLD2, as well as CDK5R1, which inactivates dynamin 1 causing vacuole retention. *Red*, direct targets of miRs-103/107; *green*, key factors; *green arrows*, upregulation; V, vacuoles; Ly, lysosomes; Nu, nucleus; m, mitochondria. Reprinted with permission from Park JK, Peng H, Katsnelson J, et al. MicroRNAs-103/107 coordinately regulate macropinocytosis and autophagy. *J Cell Biol*. 2016;215:667–685.

From a morphogenetic perspective it is well established that the limbal and corneal epithelia are fundamentally distinct (see prior reviews^24–26^). Such a diversity for two adjacent stratified squamous epithelia has made the corneal/limbal epithelia a valuable model for interrogating a variety of biologic processes. Perhaps the most consequential distinction is that the limbal epithelial basal cells are the preferential site of the corneal epithelial stem cells.^27–29^ As such, the limbal epithelium has been recognized to have major roles in maintaining corneal epithelial homeostasis, serving as the source for epithelial cells to aid in tissue regeneration following wound healing, and acting as a barrier to the egress of the conjunctival epithelium. Thus, for the past three decades much research has been directed toward understanding the structural, biochemical, molecular, and cell biological aspects of the limbal versus corneal epithelial basal cells and their microenvironments (niches). Broad conclusions drawn from these studies are that limbal epithelial basal cells are less differentiated than corneal epithelial basal cells^28^; have a subpopulation of cells that are mitotically quiescent^27^; exhibit a greater proliferative capacity than corneal epithelial basal cells^30^; express distinct cell proteins, such as transporters,^31,32^ transcription factors,^33,34^ and keratins,^28,35^ to name a few; are regulated by microRNAs that are distinct from corneal epithelia basal cells^36–40^; and are supported by a unique basement membrane/stromal interface.^26,41–43^ Despite this impressive examination of limbal versus corneal epithelial basal cells, negligible attention has been directed to macropinocytosis and autophagy, cellular processes recently demonstrated to be coordinately regulated (Fig. 3).^44^

## Regulation of Macropinocytosis and Autophagy in Limbal Epithelial Basal Cells

During studies focused on defining the microRNA (miRNA) expression profiles in limbal versus corneal epithelial basal cells, we reported that the miRs-103/107 family, which was preferentially expressed in the limbal epithelium,^37^ targeted *p90RSK2* to arrest cells in the G0/G1 phase of the cell cycle, thereby contributing to quiescence; *Wnt3a* and *MAP3K7* to increase proliferative capacity; *NEDD9*, which mediates degradation of E-Cadherin, leading to a loss of cell–cell contacts; and *PTPRM*, which controls gap junctions via repression of Connexin-43, which is a feature of stem cell-enriched epithelia.^37,45^ Collectively these findings indicated that miRs-103/107 regulate key processes associated with stem cell behavior.

We investigated the global effects of miRs-103/107 on gene expression in an unbiased manner using antagomirs-103/107 to knock down endogenous miRs-103/107 in human limbal epithelial keratinocytes (HLEKs) and conducted an mRNA profiling study.^45^ In this analysis, apoptosis, metabolic processes and response to stress were major biological events predicted to be affected by this miRNA family.^37^ Interestingly, we noted that HLEKs deficient in miRs-103/107 rapidly developed large vacuoles that originated, in part, from a dysregulation in macropinocytosis.^44^ miRs-103/107 regulate macropinocytosis, in part, at two stages. First, by targeting *NEDD9* and *SHC3*, Src, and Ras activation are attenuated and the initiation of macropinocytosis is blocked. Secondly, by targeting *ANKFY1* (*Rabankyrin5*), miRs-103/107 also interferes with the formation of the macropinosome, which contributes to the large vacuoles (Fig. 3). It is important to recognize that these target genes, while functioning in macropinocytosis, also can impact other cellular processes.

Once formed, why were the large vacuoles retained and not recycled back to the membrane? The morphology of the vacuoles was similar to autophagy-related structures. Therefore, we considered the possibility that a defect in autophagy was involved in their retention. LC3 is a marker commonly associated with autophagosomes^46^ and Rab11 is a marker of autophagosome maturation.^47^ Both of these markers colocalized with Lysotracker (a lysosomal marker) on the large vacuoles, strongly implicating an involvement with autophagy. Pharmacologic and genetic studies strongly suggested that large vacuole retention was due to a defect in the end stages of autophagy and that miRs-103/107 functioned to positively regulate this aspect of autophagy. This provided us with an opportunity to unravel the mechanisms underlying the end-stage defect. In a series of experiments, we focused on the dynamin/AP2/clathrin complex since this combination has been implicated in lysosome reformation and clearance.^48–50^ Dynamin 1 is a GTPase enzyme that functions in endocytosis, lysosomal reformation, and vacuole clearance. When dynamin is phosphorylated, phospholipid binding is blocked, effectively attenuating endocytosis.^49,50^ Loss of miRs-103/107 inactivated (phosphorylate) dynamin, which resulted in vacuole retention. This occurred via two pathways: (1) miRs-103/107 target *CDK5R1*,^51^ which is an activator of CDK5. CDK5 is a kinase for dynamin^52^ and, thus, inactivates (phosphorylation) this protein. By targeting *CDK5R1*, miRs-103/107 enable dynamin to be activated. (2) miRs-103/107 target *PLD1* and *PLD2* which downregulate phosphatidic acid (PA) and diacylglycerol (DAG) synthesis and diminishes protein kinase C (PKC) activity.^53^ Such attenuation of PKC activity dephosphorylates (activates) dynamin enabling proper end-stage autophagy. Collectively, our findings reveal that miRs-103/107 coordinately suppress macropinocytosis and preserve end-stage autophagy, which is the first demonstration that these two processes can be linked (Fig. 3). The only other connection between autophagy and macropinocytosis has been the observation that autophagy-related proteins (LC3, ATG5, ATG7, and a class III PIP-3-kinase) can be recruited to macropinosomes and phagosomes.^54^ However, these investigators suggested that macropinocytosis and autophagy were independent. The significance of the commonalities between macropinocytosis and autophagy needs further investigation.

## The Biological Significance of Autophagy and Macropinocytosis in Corneal/Limbal Epithelia

Previous work on autophagy and the ocular surface revealed 15 autophagy-related proteins associated with ocular pathology (see prior review^9^); however, many of these proteins are associated with lysosomal storage disease and/or in keratoconus corneas. Consequently, their involvement in limbal/corneal epithelial physiology is questionable. An exception is recent studies in cultured human corneal epithelial cells, demonstrating that lacritin, a tear-derived epithelial mitogen,^55^ acetylates FOXO3.^56^ Such acetylation results in a coupling with the autophagy-related protein 101 (ATG101) and the subsequent initiation of autophagy.^56^ Lacritin-induced stimulation of autophagy appears to be a relatively rapid and transient event^56^ having the potential to enable corneal epithelial cells to quickly respond to stress.

Preferential expression of a miRNA family in the limbal epithelium that is involved with maintenance of autophagy implies that this digestive process may differ in the limbal versus corneal epithelium. Using mice that transgenically express a green fluorescent protein (GFP)-labeled LC3 to assess autophagy,^57^ we noted that limbal epithelial basal cells had significantly greater amounts of LC3-positive puncta than corneal epithelial basal cells^44^ suggesting greater autophagic activity in limbal basal cells. Since autophagy is essential for survival, having a miRNA family that maintains proper end-stage autophagy, preferentially expressed in the stem cell-enriched limbal epithelium makes excellent biological sense. This is particularly germane to stem cells, which require active elimination of unnecessary proteins and organelles that accumulate during their quiescence. It is well established that a subpopulation of stem cells in the limbal basal epithelium results in this tissue having a high proliferative capacity.^27,29^ Since numerous studies have shown a positive relationship between autophagy and stem cell proliferative capacity,^7,58–61^ we investigated whether modulating autophagy affected the proliferative status of the limbal epithelium. Indeed, HLEKs treated with the autophagy inhibitor Bafilomycin, showed a marked decrease in the ability to form holoclone colonies, which is an accepted marker of proliferative capacity.^37,62^ Furthermore, we studied the corneal epithelial wound-induced proliferative response of mice deficient in Beclin 1, which is required for the early stages of phagophore formation,^13^ and noted a significant reduction in cells in the S phase of DNA synthesis in these mice, compared to littermate controls. This suggests that autophagy may be a necessary component for activation of the limbal epithelial stem cell/transit amplifying cell populations. Autophagy also may explain, in part, why stem cell-enriched epithelia, such as the limbal epithelium and the bulge region of the hair follicle, are relatively connexin 43 (Cx43) poor.^63–65^ A recent study demonstrated that Cx43 is degraded by the autophagy machinery.^66^ Our observations that autophagy is enhanced in the basal limbal epithelial cells,^44^ plus the fact that miRs-103/107 are preferentially expressed in the limbal epithelium,^37^ is consistent with the idea that this miRNA family might regulate Cx43 expression by positively regulating autophagy and targeting the protein tyrosine phosphatase receptor type M (PTPRM), which negatively regulates connexin 43-based gap junctions.^37^

Less clear is the biological significance of macropinocytosis in corneal/limbal physiology. Since the limbus is highly vascularized, we speculated that the epithelial cells may not need macropinocytosis as a means of obtaining nutrients and, thus, miRs-103/107 serve to prevent dysregulation of this process. Additionally, as stem cells are believed to be relatively quiescent, their energy needs might not be as extensive as the more differentiated corneal epithelial cells, hence a minimal requirement for rapid uptake of fluids. Our work indicated that dysregulation of miRs-103/107 in HLEKs or the limbal-derived corneal cell line (hTCEpi) leads to a rapid and massive induction of macropinocytotic-derived vacuoles concomitant with ineffective protein metabolism.^44^ Human limbal epithelial keratinocytes and hTCEpi seem to tolerate such large vesicles, as we did not detect evidence of cell death or necrosis in these cells. However, macropinocytotic-derived vacuoles in other cell types and cell lines have been associated with cell death.^17,67^ Whether limbal keratinocytes are more resistant to macropinocytotic-induced cell death is not apparent. Nonetheless, our work and that of others^17,44,67^ indicates that the induction of macropinocytosis also can negatively affect cell metabolism and cell survival when macropinosomes are involved in vacuole formation.

## Ingestion/Digestion: Gaps in Knowledge From an Anterior Ocular Surface Perspective

### Ingestion (Macropinocytosis)

As macropinocytosis has largely been ignored in the anterior ocular segment, opportunities abound for fruitful research. We speculated above on why the highly vascularized limbal stroma might not require the ability of the epithelium to internalize large quantities of solutes. The question remains does the corneal epithelium participate in and/or require active macropinocytosis? The lack of miRs-103/107 in the corneal epithelium implies that regulation against massive or dysregulated macropinocytosis is absent. It is tempting to think that the avascular nature of the corneal stroma might require a macropinosomal mechanism for nutrient uptake by the corneal basal cells. However, the dogma is that the adult resting corneal stroma is relatively desiccated, particularly in the anterior portion,^68,69^ which would argue against macropinocytosis in corneal basal cells. Another source of corneal epithelial nutrients is the tears, which supply oxygen, as well as immunologic and growth factors that are critical for epithelial homeostasis and repair. Conceivably, macropinocytosis could be a means by which superficial and wing cells “gulp” tear fluid for nutrition thereby bypassing the tight junctions, which serve as a barrier to the diffusion of molecules by sealing the intercellular space.^70^ Another role for macropinocytosis might be in response to corneal perturbations. For example, following wounding the corneal epithelium becomes compromised and one consequence is stromal swelling.^71^ In such a scenario, newly reepithelialized corneal epithelial cells might use macropinocytosis to uptake excess stromal fluid and thereby aid in stromal restitution.

Another potential role for macropinocytosis revolves around interactions with pathogens on the corneal epithelial surface. We hypothesize a possible involvement of EphA2 with macropinocytotic activation leading to corneal infections. This idea is based, in part, on the observation of EphA2 expression on the surface of corneal epithelial cells.^72^ The EphA2 receptor is a member of the Eph receptor tyrosine kinase family and in many tissues upon interacting with its ligand, ephrin A1, has been implicated in regulating proliferation, differentiation, migration, and boundary formation.^73^ Specifically in the corneal epithelium, the Eph/ephrin complex affects migration.^72^ Importantly, in other tissues and cancer cell lines, the EphA2 receptor has been implicated as a positive inducer of macropinocytosis.^23,74^ For example, the malarial parasite *Plasmodium's* P36 protein activated the extracellular ligand-binding region of the hepatocyte EphA2 receptor, which was central in the formation of a protective vacuole made of hepatocyte membrane.^74^ Macropinocytosing antibodies targeting EphA2 have been shown to be effective in killing a panel of EphA2-positive tumor cell lines.^23^ These studies raise the question of whether corneal epithelial EphA2 may be involved in macropinocytotic events? One potential role could be in the *Pseudomonas*- or *Acanthamobea*-mediated cell death of corneal epithelial cells under certain conditions, such as tear gland insufficiency, where the ocular defense system (e.g., the surfactant in the tear fluid) is compromised.^75^ While much work has been done to identify the binding and epithelial traversal of these organisms (see prior reports^76,77^ and references therein), our understanding of the binding to specific corneal epithelial receptors is incomplete. We suggest that during colonization and adherence to the corneal surface, these organisms may cause clustering of EphA2 receptors, which can activate macropinocytosis,^78,79^ and/or secrete proteins that can activate an EphA2-mediated macropinocytotic entry into corneal epithelial cells.^74^ In support of this idea, transmission electron microscopy (TEM) of corneal epithelium from eyes of mice infected with *P. aeruginosa* revealed the presence of bacteria within membrane-bound vacuoles; whether these vacuoles arose via macropinocytosis was not determined.^80^ Given the availability of EphA2 null mice, the idea that macropinocytosis has a role in aiding pathogen entry into the cornea can be easily tested.

### Digestion (Autophagy)

With respect to digestion, while we are beginning to understand how this process is regulated in the limbal epithelium, there still are several areas that need attention. For example, knowledge of the early stages of autophagy has not been defined from either a regulatory or biochemical perspective. Does the degradation of intracellular and extracellular material follow the canonical autophagy pathway or the phagocytic pathway? There is emerging evidence for an autophagosome-independent role for autophagy proteins in lysosome fusion and turnover of extracellular substrates (see the report of Florey et al.^54^ and references therein). Since corneal and limbal epithelia have distinct physiologies, are the degradation processes similar for both epithelia? As mentioned previously, based on LC3 expression, corneal epithelial basal cells appear to be less active than their limbal counterparts.^44^ In contrast, high expression of LC3 was noted in the corneal wing and superficial cell layers. These regions are in relatively close approximation to the surface and are the first areas to experience environmental stresses. Thus, it is not surprising that autophagy would be active in wing and superficial cells. Another consideration is the idea that autophagy might have a role as a mediator of early differentiation. In many tissues, autophagy is highly active during differentiation (see the study of Mizushima and Levine,^81^ and references therein). Thus, we reason that the marked LC3 expression in superficial cells may, in part, reflect a role for autophagy-related proteins as initiators of early differentiation in these cells. When HaCaT cells (a keratinocyte cell line) were induced to differentiate, release of Beclin 1 and enhancement of ATG12 and LC3II were noted, suggestive that autophagy might have a role in the early stages of differentiation.^82^ Recently, the molecular machinery involved in the removal of nuclei (nucleophagy) during the end-stages of keratinization have been detailed and this form of autophagy was demonstrated to be induced when keratinocytes differentiate.^83,84^ Taken together these observations raise questions of how autophagy is regulated in the corneal epithelium and the role of autophagy and nucleophagy in corneal epithelial differentiation. We have preliminary evidence implicating a regulatory role for miR-184, which is the most highly expressed corneal epithelial miRNA.^38^ miR-184 was initially shown to function in the corneal epithelium by inhibiting miR-205, which targeted the tumor suppresser *SHIP2*.^40^ By preserving SHIP2 levels in the corneal epithelium, proper Akt signaling was assured, which maintained corneal epithelial survival.^40^ More recently, miR-184 was shown to have angiostatic properties and, thus, functioned in maintaining corneal avascularity.^85,86^ Other functions for miR-184 in the corneal epithelium have been lineage specification,^39^ controlling familial severe keratoconus as well as cataract formation.^87,88^ We now have evidence (unpublished observations) that miR-184 targets the Nogo-B receptor (NUS1). The Nobo-B receptor stabilizes Neimann-Pick Type C2 protein (NPC2),^89^ which is required for the proper clearance of autophagosomes and, thus, has a role in regulation of autophagy-lysosomal activity.^90,91^ We posit that miR-184 targeting of *NUS1* leads to a failure of NPC2 to maintain proper autophagic flux, which is detected by a decrease in LC3 activity in corneal epithelial basal cells.^44^ In support of this idea is the reciprocal association of miR-184 and LC3 expression in the corneal epithelium. miR-184 is primarily detected in corneal epithelial basal cells, with little expression in wing and superficial cells.^38^ Conversely, LC3 pucta are low to absent in basal cells and high in wing and superficial cells. It is important to remember that all cells have autophagic capability, including corneal epithelial basal cells. A major gap in our knowledge is the mechanism(s) that corneal epithelial basal cells use to invoke autophagy during normal and pathologic conditions.

## Conclusions

The corneal/limbal epithelial system has enhanced our understanding of the biology of epithelial stem cells and their transient amplifying cell progeny, due, in part, to the distinct morphogenetic characteristics of these adjacent tissues. We believe there is an equal opportunity for similar advances to be made in our fundamental knowledge of autophagy and macropinocytosis using the corneal/limbal epithelia as a model. For example, we showed that aspects of autophagy and macropinocytosis are regulated by miRs-103/107 and others have shown biochemical commonality.^54^ The biological significance of this interrelationship requires a more in depth interrogation. It is apparent that aspects of each of these processes are regulated differently in the corneal and limbal epithelia. We also know that within either the limbal or corneal epithelium the activity of these processes may vary from basal to the outermost cells. Many of the observations have been made using submerged culture systems. While this has been a logical starting point, we live in a three-dimensional world and much work must be done using 3D-organotypic raft culture systems and/or mouse models. Ultimately, our concepts concerning autophagy and macropinocytosis must be investigated in the context of common pathologies of the corneal/limbal epithelia, such as pathogenic infections, dry eye, aberrant wound healing, and diabetic keratopathies. With the library of mouse models for human disease steadily increasing, we anticipate answers to many of these questions in the not too distant future.
